# A Low-Noise Direct Incremental A/D Converter for FET-Based THz Imaging Detectors

**DOI:** 10.3390/s18061867

**Published:** 2018-06-07

**Authors:** Moustafa Khatib, Matteo Perenzoni

**Affiliations:** Center for Materials and Microsystems, Integrated Radiation and Image Sensors Group, Fondazione Bruno Kessler, Trento 38122, Italy; perenzoni@fbk.eu

**Keywords:** readout circuit, field-effect transistor (FET), terahertz imaging, direct detectors, incremental ADC, flicker noise, lock-in, chopper, parametric amplifier

## Abstract

This paper presents the design, implementation and characterization results of a pixel-level readout chain integrated with a FET-based terahertz (THz) detector for imaging applications. The readout chain is fabricated in a standard 150-nm CMOS technology and contains a cascade of a preamplification and noise reduction stage based on a parametric chopper amplifier and a direct analog-to-digital conversion by means of an incremental ΣΔ converter, performing a lock-in operation with modulated sources. The FET detector is integrated with an on-chip antenna operating in the frequency range of 325–375 GHz and compliant with all process design rules. The cascade of the FET THz detector and readout chain is evaluated in terms of responsivity and Noise Equivalent Power (NEP) measurements. The measured readout input-referred noise of 1.6 μV${}_{rms}$ allows preserving the FET detector sensitivity by achieving a minimum NEP of 376 pW/$\sqrt{\mathrm{Hz}}$ in the optimum bias condition, while directly providing a digital output. The integrated readout chain features 65-dB peak-SNR and 80-μW power consumption from a 1.8-V supply. The area of the antenna-coupled FET detector and the readout chain fits a pixel pitch of 455 μm, which is suitable for pixel array implementation. The proposed THz pixel has been successfully applied for imaging of concealed objects in a paper envelope under continuous-wave illumination.

## 1. Introduction

Recent advancement in THz technology for developing imaging systems has drawn a great deal of attention [1,2]. Imaging at THz frequencies provides many interesting and peculiar properties, such as the non-ionizing nature and the low photon energy of the radiation, making it an inherently safe technology, a relatively high resolution (with respect to microwaves) and, lastly, the capability of penetrating several materials such as clothes, plastics and paper. Accordingly, THz radiation holds great promise for a vast variety of commercial applications ranging from security screening of concealed threats [3] to biomedical diagnosis [4] and from food inspection [5] and quality control [6] to non-destructive material testing [7].

Therefore, the recent research was devoted to the development of efficient detectors that could achieve a room-temperature performance comparable to commercially available detectors and compatible with industrial CMOS technologies. THz direct detectors were prominent, since they have successfully demonstrated adequate detection performance and modest sensitivity without using heterodyne techniques that require a complex experimental setup [8]. Among the good candidates of THz direct detectors, microbolometers [9], Schottky Barrier Diodes (SBDs) [10] and Field-Effect Transistors (FETs) [11] have been customized to build THz Focal Plane Arrays (FPAs) for imaging applications.

Typically, microbolometers require specialized process technologies, such as a post-CMOS micromachining, or above-IC wafer processing to make them compatible with CMOS technologies. Moreover, they need to operate in a particular environment (e.g., vacuum packaging) to achieve their typical sensitivity, which dramatically increases the fabrication complexity and cost. On the other hand, CMOS-based THz detectors such as SBDs and FETs are more suitable for cost-effective, video-rate imaging, thanks to CMOS technology that facilitates system-on-chip solutions through a standard fabrication process with high integration capabilities. SBDs were originally used for microwave detection, because of their high sensitivity and their ability to operate at ambient or cryogenic temperatures. However, they still have the issues of non-availability as a standard cell in CMOS and the need to bias them with a current, which directly increases the 1/*f* noise. In addition, their fabrication process still suffers from significant performance fluctuations when realized in large imaging arrays [12]. Instead, FETs have the advantage that they are not limited by their cut-off frequency as in SBDs due to the plasmonic behavior that takes place inside the transistor’s channel enabling the THz detection.

The major challenges for FET-based THz detectors are related to the low detected signal intensity, which is of the order of a few tens of microvolts with a limited bandwidth, since the frame rate goes from several tens of Hz to a few kHz in the case of THz imaging applications. With this signal characteristic, the low-frequency flicker noise and the DC-offset severely influence the FET detector sensitivity and therefore degrade the dynamic range of the imaging system. However, this can be addressed by amplifying the weak FET signal, while suppressing the flicker noise and the DC-offset and, hence, achieving a high Signal-to-Noise Ratio (SNR) within the detector’s signal bandwidth.

A standard technique to reduce the noise level consists of externally interfacing the FET detector with a lock-in amplifier for measuring its weak DC voltage signal. However, this technique is not a feasible approach for simultaneous readout of multi-pixel FET detector arrays with pixels acquiring instantaneously the signal at the kHz-frame rate. Instead, a CMOS-based readout interface can be potentially integrated inside the pixel for performing the signal amplification and filtering.

In the literature, several approaches for the readout interface have been presented, attempting to enhance FET detector sensitivity. In [13], a 3 × 5 FPA for imaging at 650 GHz has been presented, with a readout circuit realizing a differential amplifier, which was a source of significant noise and therefore degraded the system performance. Moreover, THz characterization of such an FPA is still dependent on the lock-in technique to obtain raster-scanned THz images with very slow frame rates. The work in [14] presented an architecture for a FET-based 1-k pixel camera for video imaging. In this architecture, an integration capacitor of 8 pF per pixel is utilized to decrease the integrated noise by reducing the system cut-off frequency, but still not filtering all the noise down to the signal bandwidth. Other work in [15] efficiently addressed the noise filtering by using a demodulation chain based on high-Q Switched-Capacitor (SC) filtering. However, the 1/*f* noise reduction depends on the possibility of modulating the THz source at hundreds of kHz, which is not a cost-effective solution for THz imaging system. A more recent work in [16] presented an FPA for imaging at 860 GHz, integrated with a single-readout chain containing a cascade of a chopper instrumentation amplifier and a sigma-delta ADC. Despite the achieved THz performance parameters of the imaging system, the large area of the readout circuit makes it not be compact to be fully integrated inside each pixel for simultaneous readout of multi-pixel imaging arrays. Moreover, the power consumption is expected to be large due to the noise constraints with such a complex readout structure.

In this work, we present the design and complete THz characterization of a low noise readout chain integrated with a FET THz detector, capable of preserving its minimum NEP at a low modulation frequency (<1 kHz). The proposed design aims, in terms of size and power consumption, to constitute a building block for a future focal-plane array implementation. A preliminary characterization of the proposed structure was previously presented in [17,18]. In Section 2, the design considerations for the FET THz detector are explained. Section 3 describes the architecture and circuit design of the proposed readout chain. Section 4 presents the implementation of the THz pixel structure. Moreover, the THz characterization and imaging setups along with the experimental results are discussed. Lastly, Section 5 presents the conclusions of the work.

## 2. Antenna-Coupled FET Detector

### 2.1. FET-Based THz Detector

The FET THz detection mechanism has been clarified by plasma wave theory, in which the transistor channel is modeled as an electron gas with a hydrodynamic behavior [19]. Then, the distributed resistive self-mixing theory [20] modeled the transistor channel as a Non-Quasi-Static (NQS) R-C ladder network in a more circuit-focused understanding. In both theories, the FET transistor performs a self-modulation, therefore rectifying the incident THz radiation induced by the integrated antenna into a DC signal at its drain terminal. A DC gate bias voltage is also provided to control the modulation inside the transistor channel.

**Figure 1:**
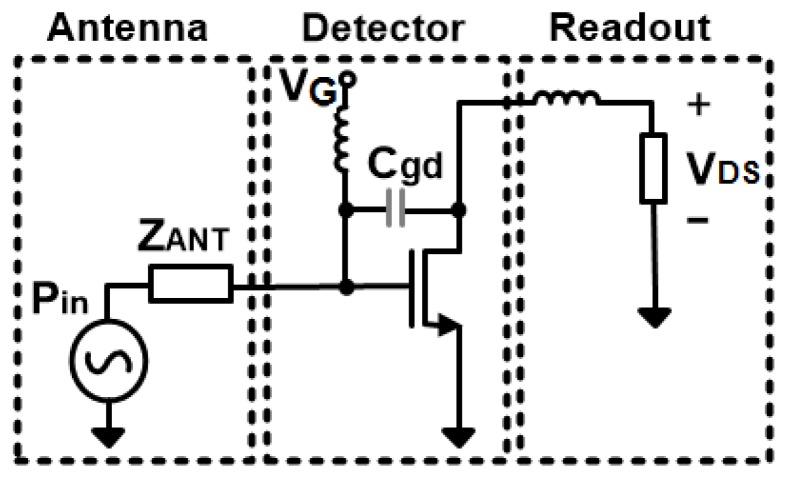
FET-based THz detector model.

**Figure 2:**
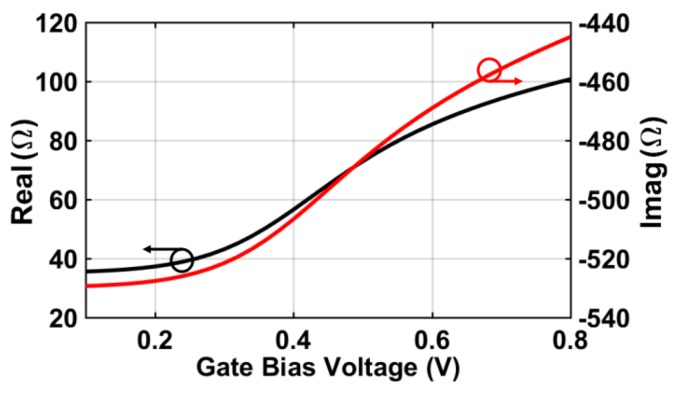
Simulated FET input impedance versus the gate bias voltage at 325 GHz.

The FET detector performance is mainly described by two key indicators, which can be clarified by Figure 1. First is the detector responsivity (*R${}_{V}$*, V/W), which is essentially dependent on the power coupling efficiency from the integrated antenna, as well as the antenna-detector impedance-matching condition. Both are highly related to the effects of parasitic elements between the gate and the drain under certain gate bias voltage. It is observed in [21] that, by designing the FET with minimum dimensions and tuning its gate voltage to reach an optimum bias point, a considerable improvement in the responsivity can be achieved at the proper impedance matching. Figure 2 shows the FET input impedance as a function of the gate bias voltage. The FET impedance has been calculated as described in [22], according to the process model parameters in the chosen CMOS technology at 325 GHz. While it is true that *R${}_{ds}$* decreases with high *V${}_{gs}$*, the real part of the FET impedance increases with *V${}_{gs}$* according to the given model because it is based on a combination of parallel/series connections and it does not necessarily have the same behavior as *R${}_{ds}$*, as shown in [16].

Next, the Noise Equivalent Power (NEP, W/$\sqrt{\mathrm{Hz}}$), which evaluates the FET detector sensitivity, is defined as the ratio between the FET’s output noise voltage spectral density (V/$\sqrt{\mathrm{Hz}}$) and the voltage responsivity *R${}_{V}$*. In a cold-biased configuration, the FET’s drain current *I${}_{D}$* is nearly negligible, and thus, the flicker noise has no relevant influence on the detector sensitivity. Thus, the FET noise contribution is mainly due to the thermal noise voltage of the channel resistance *R${}_{ds}$* as expressed by:

(1) $$V_{n}=\sqrt{4k_{B}TR_{ds}\Delta f}$$

where *k${}_{B}$* is the Boltzmann constant, *T* is the temperature and Δ*f* is the detector’s noise bandwidth. At optimum gate bias voltage, a minimum NEP can be achieved. Based on the chosen CMOS process, we anticipate that a transistor with minimum feature size (*W/L* = 0.32 μm/0.15 μm) gives a minimum NEP at *V${}_{gs}$* = 300 mV. Therefore, the channel resistance *R${}_{ds}$* = 800 kΩ at this bias point introduces a thermal noise voltage of 3.64 μV${}_{rms}$ for a detector noise bandwidth of 1 kHz. Therefore, the integrated readout chain must be designed with the input referred noise specification well below this value to maintain the FET detector sensitivity.

### 2.2. On-Chip Bow-Tie Antenna Design

**Figure 3:**
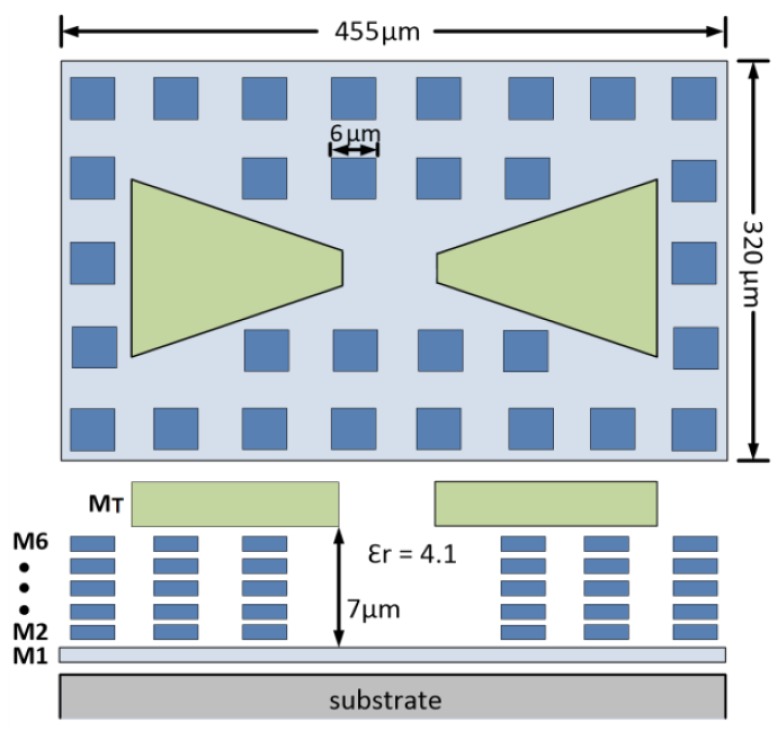
Design of the differential bow-tie antenna in the adopted 150-nm CMOS technology.

**Figure 4:**
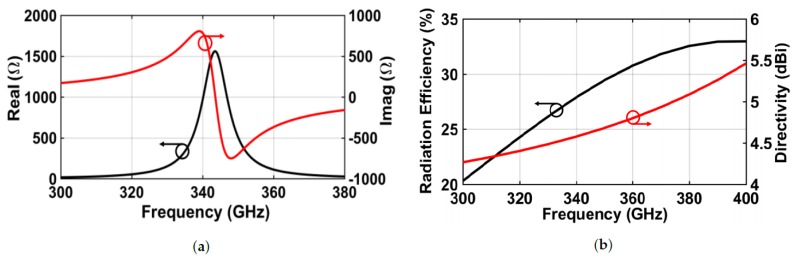
Simulation results of the bow-tie antenna: antenna impedance (**a**); antenna radiation efficiency and directivity (**b**).

A differential bow-tie antenna has been designed to operate in the frequency range of 325–375 GHz according to the methodology described in [23]. Figure 3 describes the antenna structure with the metal stack of the CMOS technology. The antenna cell dimensions are 455 × 320 μm${}^{2}$. The adopted CMOS process provides six metal layers, with a thick metal option, sandwiched between layers of a dielectric with a relative permittivity (ϵ${}_{r}$) of 4.1. The bow-tie is built by the top thick metal layer (MT), while the bottom layer M1 is utilized as a ground plane in order to shield the substrate and avoid the losses caused by the surface waves. The dielectric height from the ground plane to the bow-tie is approximately 7 μm. The remaining layers (M2–M6) are utilized as square dummy patches to achieve the required metal density in order to meet the process design rules of CMOS technology. Furthermore, the top thick metal layer (MT) is compliant with the foundry rules without any dummy because of antenna arms. The gate and source terminals of the FET detector are directly connected to the differential antenna through stacked metal vias. The antenna performance has been validated through CST Microwave studio EM simulations. In order to transfer the maximum power, the antenna impedance and the FET impedance have to be a complex conjugate pair. The antenna impedance (*Z${}_{ANT}$*) versus the signal frequency is plotted in Figure 4a, indicating $Z_{ANT}=\left(146+497j\right)$Ω at a frequency of 325 GHz. As is visible in Figure 4b, the radiation efficiency is in the range of 26–33% from 325 to 375 GHz with a range of directivity of 4.5–5.1 dBi. In fact, the obtained efficiency is low due to the thin dielectric layer between the bow-tie and the ground plane, in addition to the conductor and dielectric losses at this frequency range. However, we do not expect sidelobes due to the surface waves thanks to the reflector.

## 3. THz Readout Chain Design

**Figure 5:**
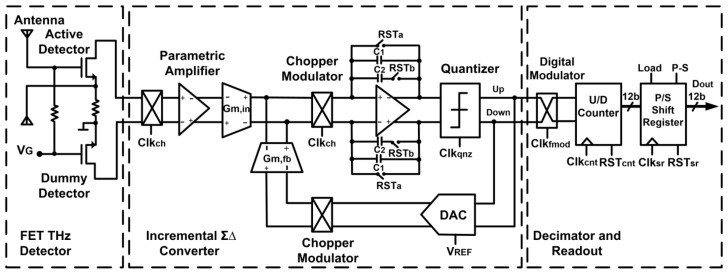
Block diagram of the proposed THz detector and readout structure.

Figure 5 illustrates the block diagram of the FET THz detector circuit integrated with the readout chain. The FET THz detector is realized by two NMOS transistors in a gate-driven configuration: One of them is connected to the integrated antenna, acting as an active FET THz detector. The second NMOS is acting as a blind detector, providing a reference voltage for offset compensation of the differential input pair, and thus, only the voltage difference between the two detectors is amplified. The two detectors are provided with the same gate bias voltage. The integrated readout chain is composed of a cascade of a preamplification and noise reduction stage based on a parametric chopper amplifier and a direct analog-to-digital conversion by means of an incremental ΣΔ converter followed by a decimation stage that provides a 12-bit digital output as a measure of the intensity of the rectified FET signal.

### 3.1. Parametric Chopper Amplification

**Figure 6:**
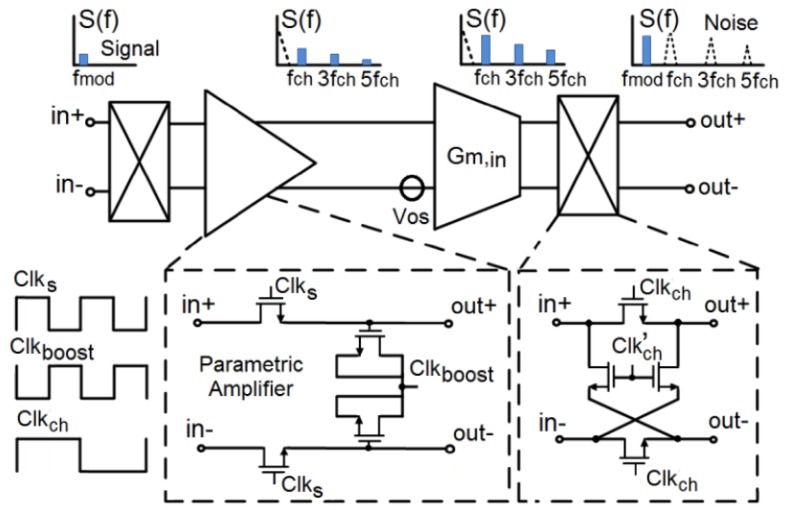
1/*f* noise and offset cancellation by using the parametric chopper amplifier.

The operation principle of the parametric amplifier is schematically clarified in Figure 6. The parametric amplifier operates in a discrete-time mode, containing two sampling switches followed by two MOS varactors [24]. The amplification process has the advantage of being noise-free with low power consumption [25]. The obtained gain is basically given as the ratio of the small-signal gate capacitances of the MOS varactors through alternating the signals *Clk${}_{s}$* and *Clk${}_{boost}$* in two non-overlapped clock phases. The parametric amplifier is enclosed by the chopper modulators that operate at a frequency of *f${}_{ch}$*, which is half of the operating frequency of the parametric amplifier. Therefore, they eliminate the 1/*f* noise from the signal path simultaneously along with reducing the thermal noise during the sampling and the boost phases of the parametric amplifier.

**Figure 7:**
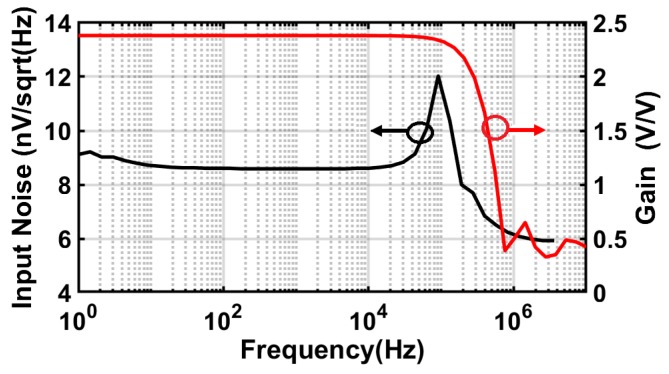
Gain and noise simulation results of the parametric amplifier at a chopping frequency of 100 kHz.

The value of the passive gain is approximately 2–3, reducing the overall input referred thermal noise contribution of the readout chain. Figure 7 shows the simulations of the gain and noise of the chopper parametric amplifier, performed by Parametric Steady-State, AC and Noise (PSS/PAC and PNOISE) analyses. The simulations show a negligible noise contribution of 9 nV/$\sqrt{\mathrm{Hz}}$ dominated by only thermal noise with a passive voltage gain of 2.3 at a chopping frequency of 100 kHz. Three choppers are placed around the parametric amplifier and the transconductors in the feed-forward and feedback paths to reduce 1/*f* noise and the DC-offset. Only the feed-forward path is considered in Figure 6 for clarification. The first chopper at the input modulates the FET signal, which is located at the source modulation frequency *f${}_{mod}$*, to the odd harmonics of the chopping frequency *f${}_{ch}$*. Then, the modulated signal together with 1/*f* noise and the DC-offset are amplified by a certain gain provided by the loop filter (G${}_{m}$ stages with the Miller integrator) of the incremental ADC. Afterwards, the last chopper at the transconductor output demodulates the signal back to *f${}_{mod}$* and shifts the 1/*f* noise and the offset to the odd harmonics of chopping frequency, such that they will be filtered out by the loop filter. All the switches in the chopper modulators are implemented with a complementary transmission gate with minimum transistor dimensions, to reduce the chopper spikes caused by the charge injection during the switching between the two clock phases.

### 3.2. Continuous-Time Incremental Conversion

Incremental sigma-delta (ΣΔ) Analog-to-Digital Converters (ADCs) benefit from the oversampling and noise shaping techniques with relaxed matching requirements similar to traditional ΣΔ ADCs [26]. The incremental ΣΔ operation offers the advantage of simultaneously integrating the rectified FET signal and averaging the detector noise, providing a one-to-one mapping between the FET signal and the digital output at each conversion cycle [27].

**Figure 8:**
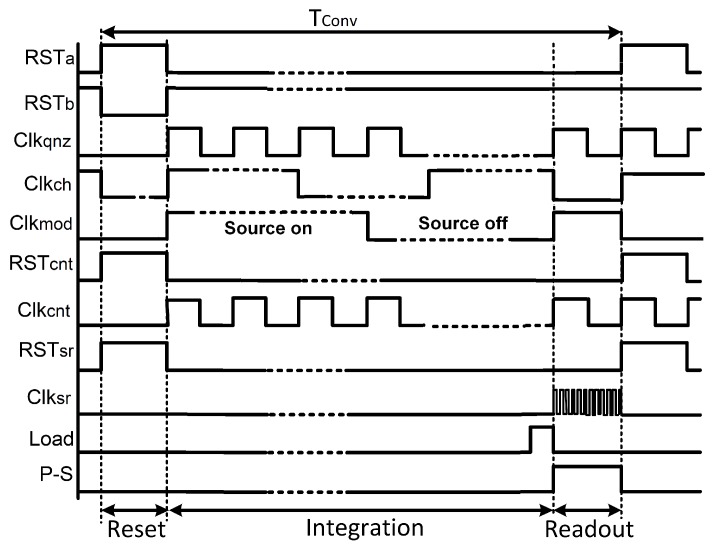
Timing diagram of the THz readout chain.

In this design, a first order modulator structure is realized, since it offers a medium resolution (i.e., more than 10 bits) that fulfills the requirements of THz imaging applications, without increasing the complexity in both the loop filter and the decimator [28]. A Continuous-Time (CT) loop filter based on a $G_{m}$-*C* structure has been implemented, since it relaxes the settling and bandwidth requirements when compared to the switched-capacitor counterpart, and hence, it achieves lower power consumption [29]. The operating principle of the readout chain is described in the conceptual timing diagram of Figure 8. The CT loop filter and the decimator are reset at the beginning of each conversion cycle (*T${}_{conv}$*). Then, the slowly varying FET voltage signal (*V${}_{FET}$*) is continuously integrated and sampled for an integration period of *T${}_{int}$* = 2${}^{n}$*T${}_{qnz}$*, where *n* is the converter resolution and *T${}_{qnz}$* is the sampling period. In each period of *T${}_{qnz}$*, the integrator output voltage is compared to the quantizer threshold and a decision is made. Next, depending on the quantizer decision, the voltage DAC feeds back either a +*V${}_{REF}$* or a −*V${}_{REF}$* signal to the input of the feedback transconductor; accordingly, the up-down digital counter, which is realized as a decimator, will either increment or decrement its value. At the end of *T${}_{conv}$*, the integrator output voltage can be described by:

(2) $$V_{int}=\frac{G_{m_{in}}}{C_{int}}T_{qnz}\left[2^{n}V_{FET}-\frac{G_{m_{fb}}}{G_{m_{in}}}\left(N_{up}-N_{down}\right)V_{REF}\right]$$

where $C_{int}=C_{1}+C_{2}$, $G_{m_{in}}$ and $G_{m_{fb}}$ are the integrating capacitor and effective transconductance values in the feed-forward and feedback paths, respectively. *N${}_{up}$* and *N${}_{down}$* are the number of subtractions and additions of *V${}_{REF}$* respectively. The Least Significant Bit (LSB) resolution is thus defined by V${}_{REF}$ scaled by both of the transconductance values in the feed-forward and feedback paths, as well as the number of clock cycles per one conversion as given by:

(3) $$V_{LSB}=\left(\frac{G_{m_{in}}}{G_{m_{fb}}}\right)\left(\frac{V_{REF}}{2^{n}}\right)$$

**Figure 9:**
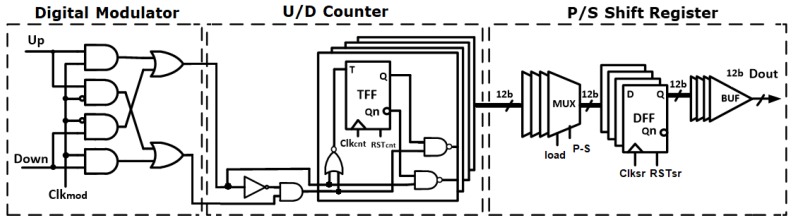
Schematic of the implemented decimator.

Finally, the digital counter value is extracted by a parallel-to-serial shift register and a new conversion cycle begins with cleared counter. The digital modulator is realized between the quantizer and the counter and operates at the same frequency of the source *f${}_{mod}$*, thus, enabling the measurement of the pixel output in both conditions (source on/source off) so as to calculate the difference between them, similar to the lock-in technique. Figure 9 shows the schematic of the implemented decimator.

**Figure 10:**
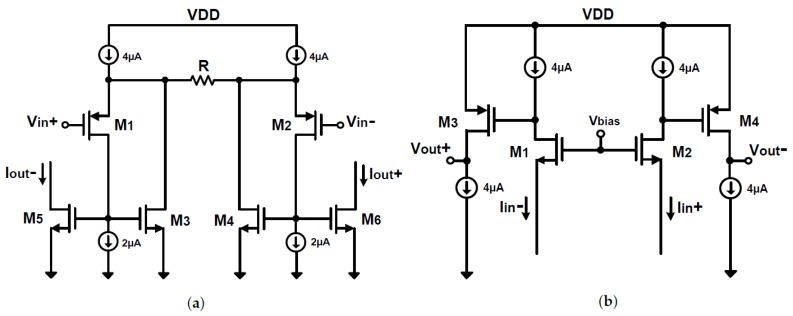
Schematic of the pseudo-differential $G_{m}$-*C* loop filter: (**a**) transconductor; and (**b**) the amplifier used in the Miller integrator.

The transconductors ($G_{m}$ stages), depicted in Figure 10a, are designed using a pseudo-differential source degeneration topology with resistors acting as transconducting elements [30]. In principle, the follower transistors (M1–M2) transfer the input voltage to the resistor, consequently improving the linearity in the V-I conversion. Then, the current mirrors (M3–M6) transfer the currents to the outputs. Therefore, the effective $G_{m}$ value will be approximately equal to 1/*R*. PMOS transistors operating in weak inversion were used for the input differential pair due to their lower 1/*f* noise contribution and to satisfy the input common-mode specification for the FET voltage signals. All the other transistors are sized to have a large length and operated in strong inversion such that the noise should be only dominated by the input pair.

The output currents from the $G_{m}$ stages are added/subtracted and then injected into a Miller integrator [31], so that the effect of finite output impedance at the transconductors’ outputs is reduced. The amplifier employed in the Miller integrator is composed of a current buffer common-gate input followed by a common-source stage as visible in Figure 10b. The bias current in the $G_{m}$ stages and the Miller integrator, which sets the noise floor of the incremental ADC, is 4 μA, resulting in a total current dissipated in the incremental loop filter of 24 μA.

**Figure 11:**
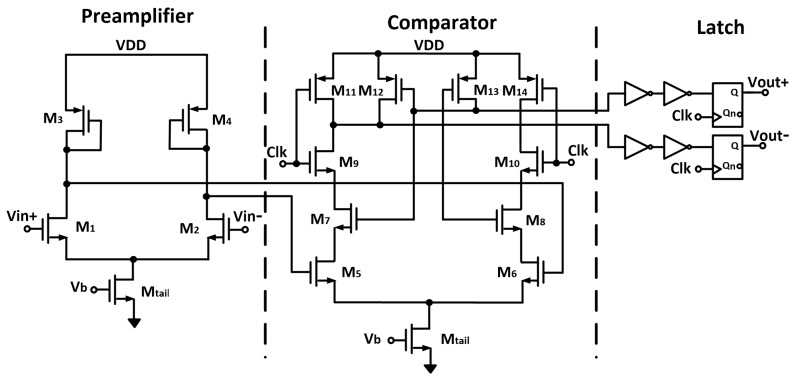
Schematic of the implemented single-bit quantizer.

Figure 11 shows the schematic of the implemented single-bit quantizer. It is based on the structure presented in [32], containing a pre-amplifier stage to avoid the kick-back noise, followed by a positive feedback latched comparator. The implemented comparator is designed with minimum transistor size, except for the bias transistor $M_{tail}$, which was scaled with respect to a current mirror-based bias circuit. Transient simulations were performed to scale the coefficients of the CT loop transfer function and guarantee that the integrator outputs are within a proper bound (i.e., $\pm V_{REF}$). The integrating capacitors are sized to be 800 fF, while the $G_{m}$ values in the feed-forward and feedback paths are scaled to be 130.33 μS and 3.33 μS, equivalent to degeneration resistors of 7.5 kΩ and 300 kΩ, respectively. The loop filter can provide an amplification for the integrated FET signal by a voltage gain that can be evaluated as,

(4) $$Gain=\left(\frac{2G_{m_{in}}}{C_{int}}\right)T_{Conv}$$

According to [33], the chopping frequency is chosen to be: (i) several times greater than the 1/*f* noise corner (which is around 10 kHz from the noise simulation), to effectively eliminate the flicker noise; and (ii) below the bandwidth of the loop filter in order to suppress the chopper spikes. The integrating capacitors are utilized as Metal-Insulator-Metal (MIM) capacitors, while high-resistivity polysilicon is used for realizing the degeneration resistors.

## 4. THz Characterization and Measurements

**Figure 12:**
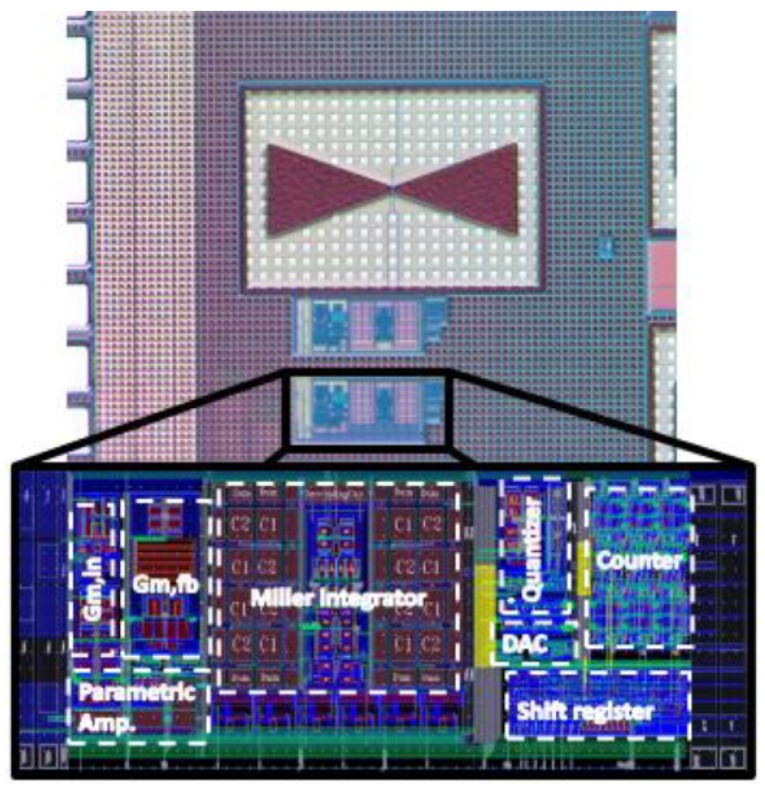
Micrograph of the fabricated THz pixel structure.

The design has been fabricated in a 150-nm standard CMOS technology. A micrograph of the pixel structure is shown in Figure 12 with an inset demonstrating the detailed layout of different blocks of the readout chain realized in a total area of 90 × 300 μm${}^{2}$. Two identical readout circuits are implemented: one of them is integrated with the antenna-coupled FET THz detector, and the other is for the purpose of performing an electrical test for the readout circuit operation.

### 4.1. Electrical Measurements of the Readout Chain

**Figure 13:**
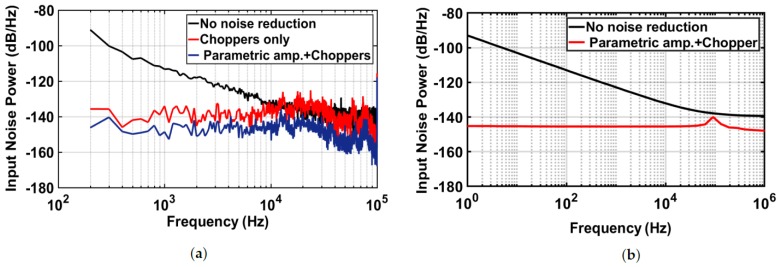
(**a**) Measured input noise power: without noise reduction (black), with the conventional chopper technique (red) and with the proposed parametric chopper amplification (blue), chopping *f* = 100 kHz; and (**b**) simulation of the input noise power of the readout chain: without noise reduction (black) and with the chopper parametric amplifier (red).

The measurement of the input-referred noise power is performed in three different configurations of the THz readout chain as presented in Figure 13a. The noise measurements were acquired at the CT loop filter output, while the differential input pins are shorted to ground. Then, it is divided by the total closed-loop gain in order to be referred to the input of the readout chain. The noise measurements are obtained at a chopping frequency of 100 kHz to effectively eliminate the 1/*f* noise and the DC-offset in the signal bandwidth.

It can be seen that the flicker noise is dominant at low frequency when the choppers are not active. In the case of bypassing the parametric amplifier and only using the conventional chopper technique, the flicker noise is effectively reduced, and only the thermal noise remains in the signal bandwidth. The input noise spectrum density averages around −139 dB/Hz at lower frequencies. However, when a passive gain of 2–3 is provided by the parametric amplifier at the beginning of the readout chain, the thermal noise is further reduced by more than 6 dB. The measured input noise remains around −146 dB/Hz, which corresponds to a total integrated noise of 1.6 μV${}_{rms}$ over a 1-kHz bandwidth. By comparing this measured value to the intrinsic noise originated by the FET detector, which is around 3.64 μV${}_{rms}$, the readout can efficiently preserve the minimum NEP of the FET detector that is only limited by its thermal noise voltage of the channel resistance. Figure 13b demonstrates the simulation of the total input noise power of the readout chain: when no noise reduction technique is applied and when the chopper parametric amplifier is operating at a chopping frequency of 100 kHz, showing a good agreement with the measurement results.

**Figure 14:**
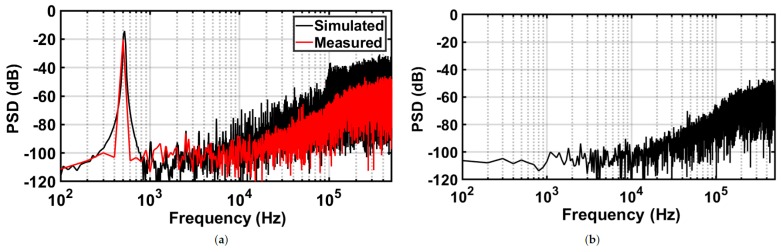
Simulated and measured output signal PSD of the incremental sigma-delta converter tested with an input sinusoidal tone at 500 Hz and sampling rate 1 MHz (**a**); and noise PSD measured with shorted input to ground (**b**).

To validate the performance of the incremental sigma-delta converter, a small amplitude 500-Hz sine wave has been injected into the readout chain. A Fast Fourier Transform (FFT) has been performed on the quantizer output by using a broadband oscilloscope. Figure 14a shows the measured graph of the output signal Power Spectral Density (PSD) showing a first order quantization noise shaping with an oversampling rate of 500, which is in a good agreement with the simulated one. Moreover, the output noise power spectral density is presented in Figure 14b. The measured SNR is 65 dB, which is equivalent to be 10.6 effective number of bits (*ENOB*), evaluated as [26]:

(5) $$ENOB=\log_{2}\left(\frac{2V_{in,max}}{V_{LSB}}\right)$$

where *V*${}_{in,max}$ is the maximum input voltage. The sampling rate of the incremental converter is set as a tradeoff between the bandwidth of the CT loop filter and the desired resolution, which depends on the number of clock cycles. Since the FET-detected signals have a maximum bandwidth of 1 kHz, a high sampling rate is not necessary. Therefore, the quantizer is designed to operate with a sampling frequency of 1 MHz to achieve an Oversampling Ratio (OSR) of 500 for the incremental conversion operation.

### 4.2. THz Experimental Setup

**Figure 15:**
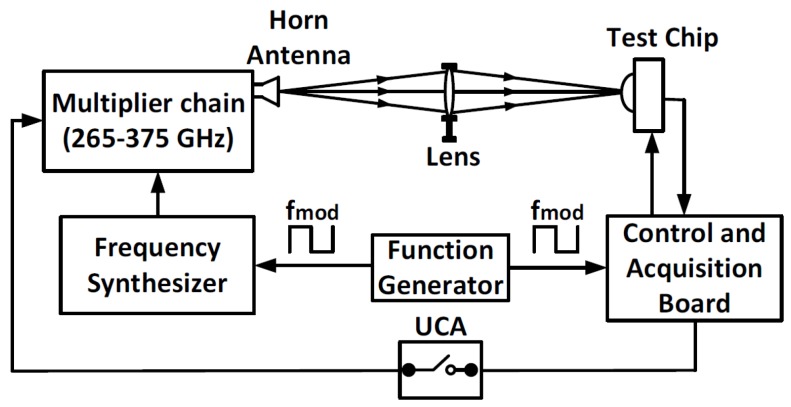
Block diagram of the THz characterization setup.

The experimental setup utilized to characterize the FET detector and the readout chain is illustrated in Figure 15. A Continuous-Wave (CW) frequency synthesizer with a multiplier chain was used as the THz source to generate a signal with frequencies between 265 and 375 GHz. Afterward, the generated signal was transmitted into free space through a horn antenna. A Zeonex lens, with a focal length of 5 cm, was used to focus the THz beam on the chip. The amplitude of THz signal was electrically modulated at different frequencies by using a function generator.

A control and acquisition board was used to generate the necessary control waveforms and to obtain the measured responses from the readout chain. In addition, a User-Controlled Attenuation (UCA) switch performed background noise subtraction measurements, hence removing any coupling or interference in the setup. According to the measurement methodology described in [34], the impinging input power received by the FET detector was measured by obtaining the impinging power density of a pyroelectric device as a reference detector with known characteristics. Then, it was multiplied by the antenna effective area *A${}_{eff}$*, as evaluated by:

(6) $$A_{eff}=\frac{D\lambda^{2}}{4\pi }$$

where *D* is the antenna directivity and λ is the wavelength of the THz signal. The impinging power delivered to the FET detector was estimated as:

(7) $$P_{FET}=\left(\frac{V_{Pyro}}{R_{pyro}}\right)\left(\frac{A_{eff}}{A_{Pyro}}\right)$$

where *A${}_{Pyro}$*, *V${}_{Pyro}$* and *R${}_{pyro}$* are the pyroelectric sensitive area, the measured pyroelectric voltage response and its responsivity, respectively.

### 4.3. Antenna-Coupled FET Detector Measurements

**Figure 16:**
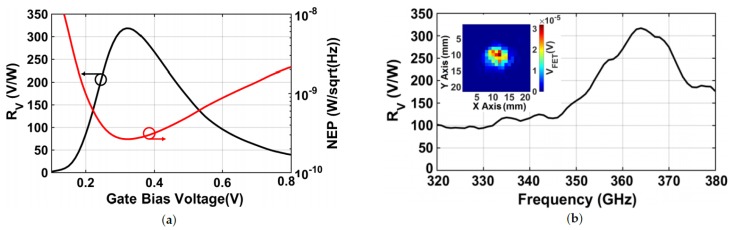
Measured FET voltage responsivity and Noise Equivalent Power (NEP) versus gate bias voltage (**a**); and measured FET voltage responsivity versus signal frequency (**b**).

**Figure 17:**
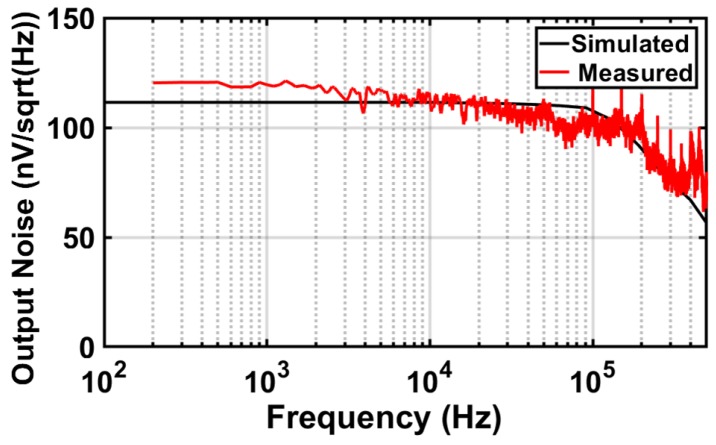
Simulated and measured FET detector noise voltage spectral density versus frequency.

Initially, the performance of a standalone antenna-coupled FET THz detector was evaluated through a lock-in amplifier with sensitivity and time constant of 500 μV and 200 ms, respectively. The FET voltage responsivity was evaluated as the measured FET voltage response *V${}_{FET}$* normalized by the impinging power *P${}_{FET}$*. By sweeping the gate bias from the sub-threshold to a strong inversion region, as is visible in Figure 16a, the FET detector achieved a peak responsivity of 318 V/W at a gate bias voltage of about 0.3 V; while it decreased towards both: (i) the strong inversion region, since the *R${}_{ds}$* was reduced, and thus, the output voltage drop across it decreases, as well, and (ii) the sub-threshold, due to the lowering of the cutoff frequency of the FET-lock-in interface well- below the source modulation frequency [35,36]. The FET detector output noise voltage spectrum density was measured around 115 nV/$\sqrt{\mathrm{Hz}}$, which was equivalent to the thermal noise contribution originated from the measured FET channel resistance *R${}_{ds}$* = 800 kΩ at a gate bias voltage of 0.3 V, as shown in Figure 17. Then, NEP can be estimated by dividing the measured thermal noise voltage by the FET responsivity, that is,

(8) $$NEP=\frac{\sqrt{4k_{B}TR_{ds}}}{R_{V}}$$

Correspondingly, a minimum NEP of 281 pW/$\sqrt{\mathrm{Hz}}$ was obtained at the same gate bias point of 0.3 V. Figure 16b shows the frequency sweep analysis for the FET responsivity with an inset illustrating the THz beam spot sensed by the FET detector. The beam spot was acquired on an *X*-*Y* transverse plane to the optical axis by scanning the test chip in 0.4-mm steps using a stepper motor. It is also possible to notice that the FET detector responsivity is above 200 V/W from 355–375 GHz with a peak value of 318 V/W near 365 GHz.

### 4.4. Readout Responsivity and NEP Measurements

Measurements of responsivity and NEP for the FET THz detector were eventually acquired through the implemented readout chain, in place of the external lock-in amplifier. The measurements were performed by modulating the THz source and the digital modulator (see Figure 5) at three different frequencies within the range of the FET detector bandwidth of 1 kHz.

**Figure 18:**
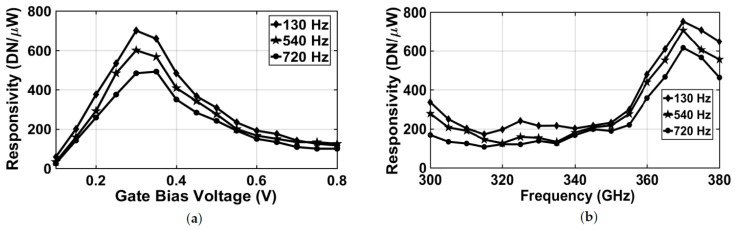
Readout responsivity as a function of FET gate bias voltage (**a**) and signal frequency (**b**).

Thanks to the digital modulator, the recorded digital response was the net value of the difference between the two states of the modulating signal waveform (while the THz signal was present and while there was no THz signal), such that it could perform the lock-in function. The voltage response of the FET detector was recorded as 12-bit digital code through the shift register output per each conversion cycle. The integration time of the readout chain was correlated to the modulation frequency of the FET signals (<1 kHz), which was typically in the range of 1–10 ms. The measured responsivity as a function of gate bias voltage is presented in Figure 18a at different modulation frequencies, showing a peak response near a 0.3-V gate bias voltage.

Since the readout directly converts the FET response to a digital output without any representative voltage except the detector’s, we need to rewrite the responsivity, which then would not be comparable to *R${}_{V}$* of course, but the NEP still gave a metric for comparison because it was input-referred. The responsivity was expressed as a digital number per unit of impinging power (DN/W) instead of (V/W), as given by:

(9) $$R_{dig}=\frac{DN}{P_{FET}}$$

**Figure 19:**
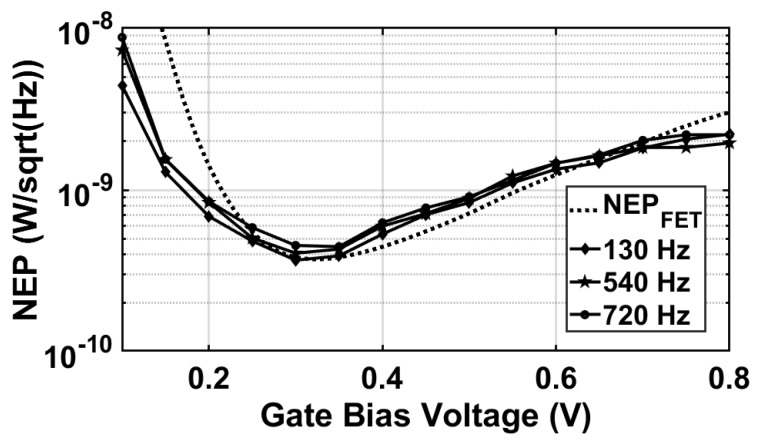
NEP as a function of FET gate bias voltage (measured at 365 GHz).

Similarly, Figure 18b shows the responsivity as a function of signal frequency, exhibiting peak values near 365 GHz. NEP curves in Figure 19 were measured by calculating the standard deviation of several acquired digital outputs $\delta_{DN}$, i.e., the RMS of the output code of each conversion cycle, divided by the measured responsivity acquired at a signal frequency of 365 GHz. Then, it was divided by the square root of the FET bandwidth (e.g., *f${}_{mod}$*), as given by:

(10) $$NEP=\frac{\delta_{DN}}{R_{dig}\sqrt{f_{mod}}}$$

The minimum NEP value of 376 pW/$\sqrt{\mathrm{Hz}}$ was obtained at a gate bias voltage of 0.3 V and a modulation frequency of 130 Hz. The measured NEP was only due to the thermal noise contribution of the FET detector without being influenced by the readout noise, since the measured input noise power was significantly below the FET thermal noise. However, due to the fact that the dummy FET detector contributes to the thermal noise voltage by its channel resistance, the obtained NEP by the readout chain was approximately $\sqrt{2}$-times higher than the measured NEP of a single FET detector. The NEP of both active and dummy FETs is visible in the dashed line in Figure 19, exhibiting a good match with the readout measurements. It can be seen that the total NEP (FETs + readout) was generating less noise than FETs only at a lower gate voltage due to the fact that the responsivity of the detector alone was lower in that range due to the RC time constant (e.g., the connection to the instrumentation), which was not present with the readout chain.

### 4.5. THz Imaging

**Figure 20:**
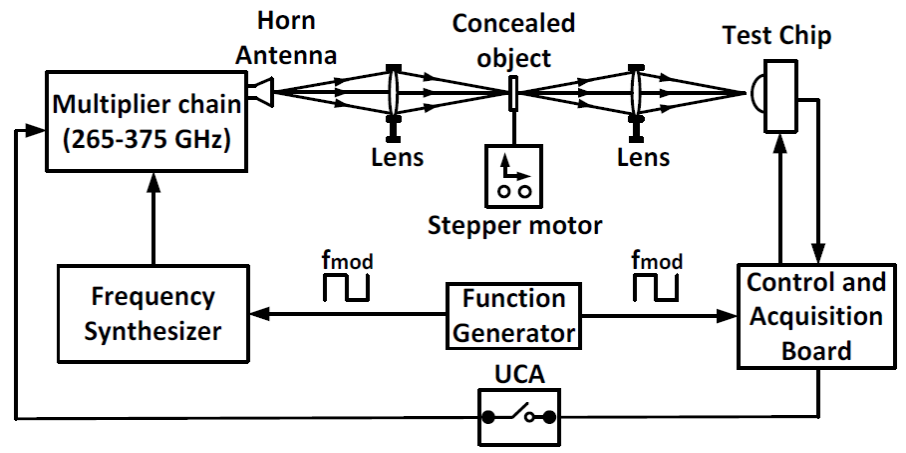
Block diagram of the THz imaging setup.

**Figure 21:**
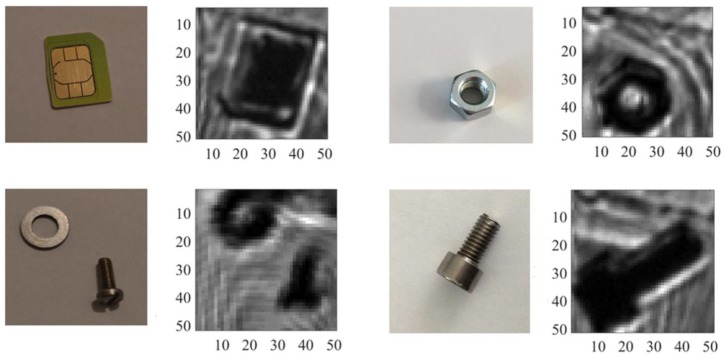
THz images of different metallic/plastic objects hidden inside a paper envelope acquired at 365 GHz (source modulation f=130 Hz) along with the photographs of the objects.

The optical experimental setup used for performing THz imaging acquisition is presented in Figure 20. The image object was vertically positioned in the focal point between two Zeonex lenses, such that the THz beam was focused on the object by the first lens and collimated by the second lens and then refocused on the test chip. Since our sensor contains only a single pixel, mechanical scanning was required to obtain a wide field of view. A stepper motor stage was used to scan the objects in the vertical and horizontal directions by a step size of 0.4 mm. Metallic and plastic objects concealed inside a paper envelope were mechanically scanned and captured in transmission mode at 365 GHz and a modulation frequency of 130 Hz. As shown in Figure 21, the scanned images clearly resolve the structural details of the objects such as screws, a SIM card, a nut and a metallic ring. The scanned area is 20 × 20 mm${}^{2}$ with a total resolution of 50 × 50 pixels.

The total acquisition time of each image was around 3.5 h, due to the limited speed of the stepper motors, which was essentially dominated by the actuator’s speed, while the effective acquisition time amounted to just several seconds. The background interference pattern appearing in the images was produced by the standing waves inside the paper envelope. In comparison with the conventional lock-in technique, typically the lock-in amplifier required an integration time in the range of 200–500 ms in order to achieve similar signal quality, while the proposed readout chain acquires the data of each pixel during an integration period in the range of 1–10 ms, depending on the applied modulation frequency. This is regardless of the time consumed by the stepper motors to move between different pixels. The overall performance of the presented THz pixel structure is summarized in Table 1 and compared to the recently-reported state of the art. The proposed THz pixel features: (1) a first order incremental ADC that is compact in terms of area and power and can be fully integrated inside the THz pixel, providing simultaneous integration and readout; (2) suitable for pixels also with smaller antennas (e.g., 800 GHz); (3) it does not require sources with a high modulation frequency, so that the cost of the THz imaging system could be significantly reduced; (4) a direct conversion to the digital domain, which means robust and easy signal management. (5) Low input-referred noise.

## 5. Conclusions

In this paper, a noise efficient readout chain integrated with an antenna-coupled FET THz detector has been developed in 150-nm CMOS technology. The electrical measurements and THz characterization demonstrate the performance of the readout chain, which effectively performs the function of integrated lock-in amplification with a direct digital output. Owing to the in-pixel parametric chopper technique and the anti-aliasing filtering of the incremental conversion loop, the flicker noise and the DC-offset are effectively eliminated without modulating the THz source at high frequencies. The integrated readout noise of 1.6 μV${}_{rms}$ over a 1-kHz bandwidth resulted in a peak-SNR of 65 dB, sufficient for obtaining a good signal quality for THz imaging applications. The detection behavior of the FET detector with the readout chain has shown a good sensitivity with a minimum NEP value of 376 pW/$\sqrt{\mathrm{Hz}}$ at 365 GHz, while providing a direct digital responsivity. Moreover, the acquired THz images through the readout chain with its compact pixel size of 455×455 μm${}^{2}$ and the low power consumption of 80 μW have demonstrated good performance as detectors in THz imaging.

## Figures and Tables

**Table 1:** Performance comparison with the recently reported state of the art.

	This Work	T-TST’17 [16]	Sensor’16 [15]	JSSC’12 [14]	JSSC’09 [13]
Process	0.15 μm	0.18 μm	0.13 μm	65 nm	0.25 μm
Input-Referred Noise	1.6 μV${}_{rms}$	2.03 μV${}_{rms}$	0.2 μV${}_{rms}$	2.45 μV${}_{rms}$	-
Power Consumption	80 μW	-	320 μW	2.5 μW	5.5 mW
Source Frequency	325–375 GHz	860 GHz	270 GHz	856 GHz	650 GHz
Modulation Frequency	10 Hz–1 kHz	177 Hz	156 kHz	5 kHz	30 kHz
On-chip Antenna	Bow-tie antenna	Patch antenna	Bow-tie antenna	Ring antenna	Patch antenna
Pixel Size	455 × 455 μm${}^{2}$	1330 × 440 μm${}^{2}$	240 × 240 μm${}^{2}$	80 × 80 μm${}^{2}$	200 × 150 μm${}^{2}$
Maximum Responsivity	783 DN/μW	3.3 kV/W	300 kV/W	140 kV/W	80 kV/W
Minimum NEP	376 pW/$\sqrt{\mathrm{Hz}}$	106 pW/$\sqrt{\mathrm{Hz}}$	533 pW	12 nW	300 pW/$\sqrt{\mathrm{Hz}}$
	@130 Hz	@177 Hz	@156 kHz	@500 kHz	@30 kHz
